# Quality of family planning counseling and associated factors among women attending family planning clinics at selected health centers in Akaki Kality sub-city, Addis Ababa, Ethiopia

**DOI:** 10.3389/fgwh.2022.939783

**Published:** 2022-12-01

**Authors:** Girma Garedew Goyomsa, Leta Adugna Geleta, Sisay Abebe Debela, Nejiba Reshid, Amana Ogeto Luke, Tafesse Lamaro Abota, Derara Girma, Hiwot Dejene

**Affiliations:** ^1^ Public Health Department, College of Health Science, Salale University, Fitche, Ethiopia; ^2^ Blood Donation, National Blood Bank Service of Ethiopia, Addis Ababa, Ethiopia; ^3^ Public Health Department, Lancha Campus, Rifty Valley University, Addis Ababa, Ethiopia; ^4^ School of Public Health College of Health Sciences, Addis Ababa University, Addis Ababa, Ethiopia

**Keywords:** family planning, quality of counseling, factors, women, Ethiopia

## Abstract

**Background:**

Ethiopia has achieved a remarkable improvement in the provision of family planning. The modern contraceptive prevalence rate has shown a fivefold increment in the last two decades, yet the family planning service in the country is still deficient and characterized by poor counseling quality.

**Objective:**

The aim of the study is to assess the quality of family planning counseling provided and the associated factors at selected health centers in Akaki Kality sub-city, Addis Ababa, Ethiopia.

**Method:**

A cross-sectional study was conducted among 678 randomly selected women attending family planning services at health centers in Addis Ababa, Ethiopia. Multivariable logistic regression analysis was performed to identify factors associated with the quality of family planning counseling.

**Result:**

A total of 678 women participated in the study. About 29.1% [95% confidence interval (CI): 25.7%–32.6%] of the respondents were adequately counseled. Age groups 37–49 [adjusted odds ratio (AOR) = 2.7; 95% CI: 1.1–6.6], being in marital union (AOR = 2.8; 95% CI: 1.2–6.7), attaining secondary education (AOR = 1.9; 95% CI: 1.1–3.6) or higher education (AOR = 2.2; 95% CI: 1.2–4.3), and visit status (AOR = 1.6; 95% CI: 1.1–2.4) were significantly associated with good counseling.

**Conclusion:**

Less than one in three women was counseled adequately. Health professionals should give due attention to younger women, single clients, and clients with their first presentation to the health facility. It also indicates that promoting education among Ethiopian women is crucial for a positive outcome of family planning counseling.

## Introduction

The World Health Organization (WHO) defined family planning (FP) as the ability of individuals and couples to anticipate and attain their desired number of children and the spacing and timing of their births (1). As of 2017, 1.6 billion women of reproductive age (15–49) live in developing regions. Among these, about 50% of them want to avoid pregnancy. However, only three-quarters (671 million) are using modern contraceptives such as injectables, implants, condoms, pills, sponges, spermicides, intrauterine devices (IUCD), and vaginal rings (2). Moreover, 27.7% of married women in sub-Saharan African countries have an unmet need for contraception (2).

Family planning counseling is a continuous process through which the FP service provider and the client explore and discuss the client's needs and FP options (3). It is one of the critical elements in the provision of quality FP services (4). Successful counseling before discharge is likely to have an impact on subsequent contraceptive uptake and maintenance. Evidently, women who are well counseled have an 80% reduced risk of family planning discontinuation (5). Likewise, family planning counseling during antenatal care (ANC) services has a significant effect on promoting postpartum modern family planning use (6). Furthermore, counseling during family planning provision reduces the unmet need for family planning by 27% (7).

Various interventions were designed and implemented to improve family planning utilization in low- and middle-income countries. The interventions include antenatal, postnatal, combined antenatal and postnatal, and integration of family planning into different services (8). Nevertheless, modern contraceptive use in sub-Saharan African countries is exceptionally low. Studies indicated that only 39% of women in need of contraceptives in the region are using them (9). In addition, the service in the region is characterized by a weak client–provider relationship (10). Furthermore, the counseling level is also inadequate; for instance, the proportion of counseled women is 56.7% in Kenya (11).

Ethiopia, Africa's second most populous country, is one of the few countries where contraceptive prevalence has doubled twice in about a decade (12). The modern contraceptive prevalence rate (CPR) among married women has also increased by nearly fivefold in Ethiopia, from 8.1% in 2000 to 37% in 2019 (13). However, efforts to move beyond a focus on contraceptive access and uptake toward quality service are very important. Yet the family planning service in the country is still deficient and characterized by poor counseling quality (14). The percentage of women who received high family planning counseling services declined since 2015 from 39% to 12% in 2019 nationally (13). Other studies in the county reported varying prevalence of adequate counseling; two separate studies conducted in Addis Ababa reported the prevalence as 28.2% (15) and 34.8% (16). A nationwide study also indicated that only 30% of women had received sufficient information during counseling (17). Even though a couple of studies have been conducted on the quality of the counseling service, they focused only on women in antenatal care and hospital settings. Evidence on the quality of family planning counseling in the family planning clinics run by health centers is therefore deficient; thus, the present study aimed to evaluate the quality of family planning counseling provided and associated factors at selected health centers in Addis Ababa, Ethiopia.

## Methods

### Study setting and design

A facility-based cross-sectional study was conducted in health centers found in Akaki Kality sub-city, Addis Ababa, Ethiopia, from May to June 2020. Akaki Kality is one of the 10 sub-cities of Addis Ababa. It is in the southern part of the city. It is characterized by the highest total fertility rate of all other sub-cities. Based on the 2022 population projection, the total population of the sub-city is 255,348 of which 131,525 (51.5%) are females. There are about 10 public health centers in the sub-city that provide family planning services for the community. All these facilities, namely, Tulu Dimtu, Akaki, Akaki Kela, Selam Fire, Kality, Saris, Sirte, Kilinto, Gelan Gura, and Gelan health centers, were included in the study.

### Population and eligibility criteria

The source population was all reproductive-age women who were attending family planning services at health facilities in the Akaki Kality sub-city, Addis Ababa, Ethiopia. The study population was selected from reproductive-age women who were attending family planning services at health facilities of Akaki Kality sub-city during the data collection period. All reproductive-age women (15–49 years) who came during the study period and were willing to participate were included in the study.

### Sample size

The sample size was calculated using the STAT CALC application of Epi-Info version 7.2.2.6. The study had two objectives, and thus, the sample size was calculated separately for both objectives, with the larger sample size being taken as the final sample size. For the first objective, it was calculated using a single population proportion approach with the assumptions of a 95% confidence interval (CI) and a 5% margin of error. Accordingly, taking the 34.8% proportion of women adequately counseled from a previous study (16), the sample size was found to be 349. For the second objective, it was calculated with the assumptions of a 95% confidence interval and 80% power. Taking the 11.5% proportion of good family planning counseling from a previously conducted study (15) and a minimum odds ratio of 2.5 to be estimated, the sample size was found to be 622. Finally, adding a 10% nonresponse rate to the largest sample size calculated, which is 622, the final sample size was 684.

### Sampling procedure

The study participants were selected using a systematic random sampling technique. First, the sample size was distributed using proportional allocation to size (PAS) to each health center in the sub-city. The individual clients (women who came for family planning service) were approached by calculating the sampling interval, *K* [*N*/*n*, where *N* is the total number of women who attend family planning clinics and *n* is the final sample size calculated]. Accordingly, the total number of women who were attending family planning clinics were *N* = 2,189, of whom *n* = 684 are included in the final sample size that yields a sampling interval (*K*) of three. The first patient to be interviewed was selected using the lottery method from among the first three clients. Finally, every three clients coming to the family planning clinics for a family planning service were included until the sample size was attained.

### Measurement

Family planning counseling quality was assessed with the GATHER approach. The tool has six counseling elements described by the acronym (Greet, Ask, Tell, Help, Explain, and Return) (18). The level of family planning counseling was derived by summing up the scores on each GATHER item; the score ranges from 0 to 25 with higher scores indicating better counseling quality. Family planning service counseling quality was defined as adequate if the score is ≥16 and otherwise inadequate. The quality of counseling was further classified as “excellent” for scores ≥24, “good” for scores 19–23, “average” for scores 14–18, “poor” for scores 10–13, and “very poor” for scores ≤9 (19).

### Data collection procedure

Data were collected using a structured questionnaire through the face-to-face exit interview. The questionnaire was adopted from previous literature (14–17, 19). The questionnaire was divided into three parts: sociodemographic characteristics of the clients, quality of family planning counseling measuring items, and reproductive health-related questions. The questionnaire was prepared in English first and then translated into Amharic and then back to English by a language expert to check the consistency. It was then pretested on 5% (34) of the respondents outside the study area and necessary modifications were taken accordingly. Data were collected by bachelor's degree holding midwifery professionals.

### Data processing and analysis

Each questionnaire was checked for completeness and consistency and was entered into Epi-Info version 7.0. Then data were exported to SPSS (Statistical Package for Social Sciences) version 24 for cleaning, editing, and further analysis. Descriptive statistics such as frequencies, tables, and figures were used to show sociodemographic characteristics and other background variables. The study also employed logistic regression analysis to identify the factors associated with the quality of family planning counseling. First, bivariate logistic regression analysis was performed for each independent variable, and then those whose *p*-values are less than 0.25 were considered candidate variables for multivariable logistic regression to control possible confounders. The backward enter method was employed in multivariate logistic regression, where adjusted odds ratios (AORs) with their corresponding CIs were used to assess the strength of the associations between dependent and predictor variables at *p*-value  ≤ 0.05 cut-off points. The model fitness was checked using the Hosmer–Lemeshow goodness fit test and declared fit. Multicollinearity was also checked at a 10% variance inflation factor, and no multicollinearity was detected.

### Ethical consideration

An ethical clearance letter was obtained from the Rift Valley University Abichu campus Ethical Review Committee with the reference number RVU-121/2020. The purpose of the study was clearly explained to the patients, and written informed consent was obtained from each participant prior to the interview. Additionally, all the information obtained from each study participant was kept confidential throughout the process of the study. Personal identifiers were not written on the questionnaire to ensure the confidentiality of the respondents.

## Results

### Sociodemographic characteristics

A total of 678 respondents participated in the survey, with a response rate of 99%. The mean (standard deviation) age of respondents was 25.86 (4.64) years. More than 4 in 10 [292 (43.1%)] of the respondents were in the 25–30 years age group. The mean (*SD*) number of children among the respondents was 1.4 (1.0). Concerning the educational status of the participants, about 290 (42.8%) of them reported that they attended only primary education. The majority of the participants [582 (85.8%)] were married. More than half [385 (56.8%)] of the respondents had previous experience of visiting the family planning clinic, while the rest [293 (43.2%)] were new clients (Table 1).

**Table 1:** Sociodemographic characteristics of women attending family planning clinics at health centers in Akaki Kality sub-city, Addis Ababa, Ethiopia, 2020 (*N* = 678).

Variables	Categories	Frequency	Percentage
Age	18–25	290	42.8
	26–30	293	43.2
	31–35	65	9.6
	≥36	30	4.4
Educational status	No formal education	114	16.8
	Primary	290	42.8
	Secondary	166	24.5
	College and above	108	15.9
Marital status	Single	96	14.2
	Married	582	85.8
Number of children	0	129	19.0
	1–2	478	70.5
	3–4	71	10.5
Visit status	New	293	43.2
	Returning	385	56.8

### Types of family planning utilized

**Figure 1:**
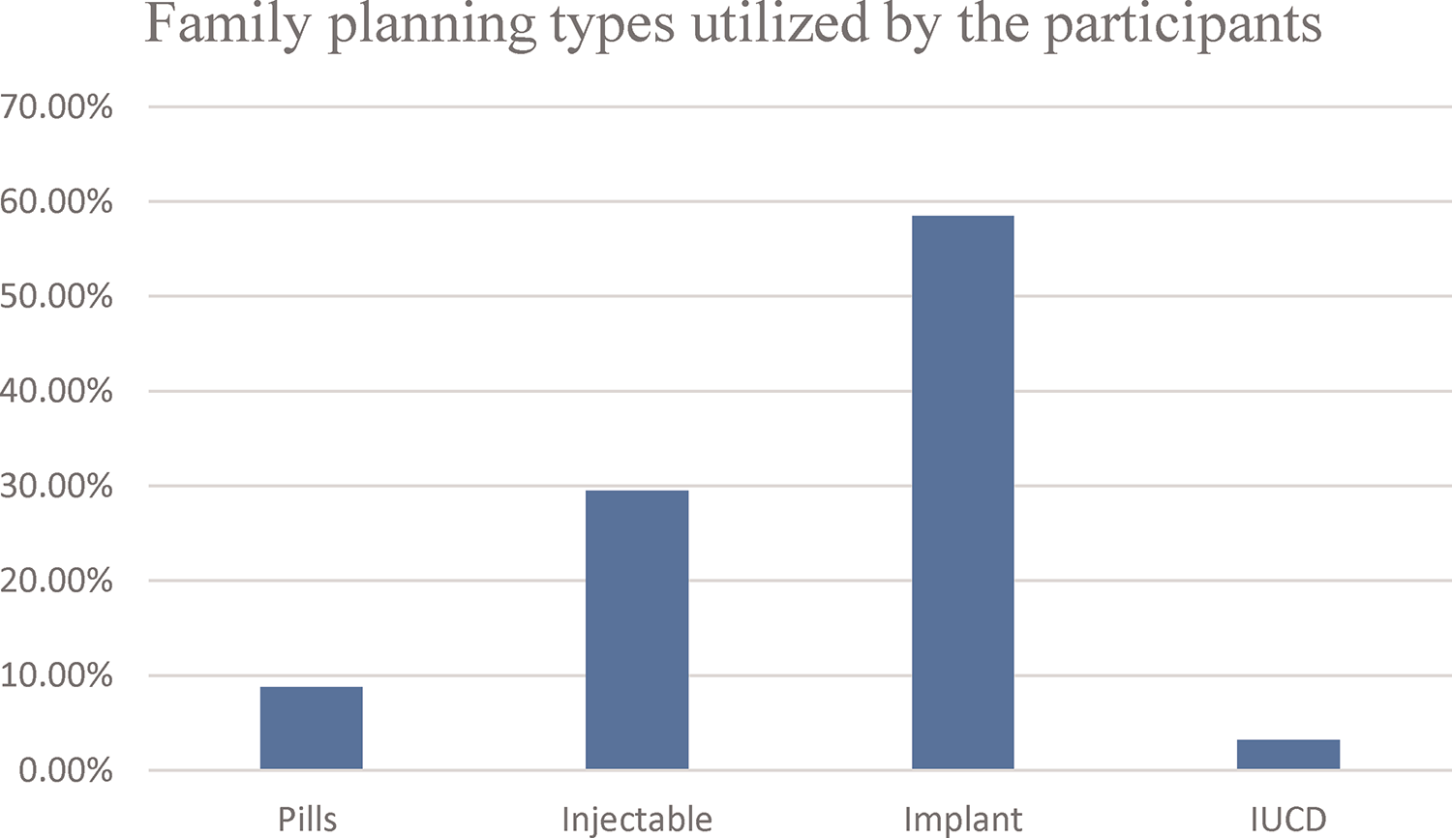
Types of family planning utilized among women attending family planning clinics at health centers in Akaki Kality sub-city, Addis Ababa, Ethiopia, 2020 (*N* = 678).

Regarding the family planning methods utilized by the participants, more than half of the study participants (61.8%) use long-acting contraceptive methods. Concerning the specific family planning methods utilized by the participants, more than half of them [396 (58.5%)] were using implants followed by injectables [200 (29.5%)] and pills [60 (8.8%)], and the rest [22 (3.2%)] were using IUCD (Figure 1).

### Quality of family planning counseling

Out of 678 study participants, none of them mentioned that their service providers introduced themselves to their clients. A substantial proportion [641 (94%)] of the providers arranged for privacy sessions during counseling whereas only 22% of the respondents reported that the service providers asked their clients about their concerns to use modern FP methods. Almost half [364 (53.2%)] of the clients reported that they were told about the benefits and risks of FP methods; the majority [565 (83.3%)] of them were told about the potential side effects. The majority [648 (95.6%)] of the clients stated that they were asked about which FP method they wanted to use. However, a small proportion [75 (11.1%)] of clients responded that service providers asked them to repeat back instructions. Nearly, 1 in 5 [127 (18.7%)] of the service providers reminded the client of the potential side effects, and 31 (4.6%) reminded the client of dangerous signs/symptoms. More than half [410 (60.5%)] of the study participants claimed that they were told about what to do if a problem occurs, and the majority [643 (94.8%)] were told when to come back (Table 2).

**Table 2:** Responses of clients about family planning counseling quality among women attending family planning services at health facilities in Akaki Kality sub-city, Addis Ababa, Ethiopia, 2020 (*N* = 678).

Variables	Yes, *N* (%)	No, *N* (%)
Greet		
Greet in a respectful manner	470 (69.3)	208 (30.7)
Introduce themself	0 (0)	678 (100)
Arrange for privacy	641 (94.5)	37 (5.5)
Ask		
Ask client's medical history	375 (55.3)	303 (44.7)
Ask clients about whether she is breastfeeding or not	505 (74.5)	173 (25.5)
Ask how much time the client and her partner live together	111 (16.4)	567 (83.6)
Ask if the client was concerned about using a modern method	152 (22.4)	526 (77.6)
Ask the client's plan to have more children	271 (40.0)	407 (60.0)
Tell		
Informs all the contraceptive methods available	526 (77.4)	152 (22.6)
Asks which method interests the client	373 (55.0)	305 (45.0)
Ask the client what already knows about the meth	175 (25.8)	503 (74.2)
Corrects incorrect in formations/myths	267 (39.4)	411 (60.6)
Describe the effectiveness of the method	144 (21.2)	534 (78.8)
Uses audio/visual aids during counseling	300 (44.2)	378 (55.8)
Describes the benefits and risks	364 (53.7)	314 (46.3)
The potential side effects	565 (83.3)	113 (16.7)
Answers the client's questions clearly	419 (61.8)	259 (38.2)
Help		
Asks if anything is not understood	189 (27.9)	489 (72.1)
Asks what method do you want	648 (95.6)	30 (4.4)
Explain		
Clearly explain instructions on successful using methods	452 (66.7)	226 (33.3)
Asks the client to repeat back instructions	75 (11.1)	603 (88.9)
Reminds the client of the potential side effects	127 (18.7)	551 (81.3)
Reminds the client of dangerous signs/symptoms	31 (4.6)	647 (95.4)
Explain what to do if problems occur	410 (60.5)	268 (39.5)
Return		
Plans a revisit schedule for the client	643 (94.8)	35 (5.2)

### Level of family planning counseling

The mean (*SD*) score of family planning counseling was 12.14. The proportion of women who had inadequate family planning counseling was 70.9%; 95% CI 67.4%–74.3%. Further classifying the levels of quality of family planning counseling, about 224 (33.0%) were at average level followed by very poor [211 (31.1%)], poor [198 (29.2%)], good [45 (6.6%)], and none of the counseling services was excellent (Table 3).

**Table 3:** Levels of family planning counseling quality among women attending family planning services at health facilities in Akaki Kality sub-city, Addis Ababa, Ethiopia, 2020 (*N* = 678).

Levels of family planning counseling	Frequency	Percentage
Excellent	0	0
Good	45	6.6
Average	224	33.0
Poor	198	29.2
Very Poor	211	31.1

### Factors associated with quality of family planning counseling

After adjusting for possible confounders, the age of respondents, marital status, educational status, and familiarity with the family planning clinic were significantly associated with the quality of family planning counseling. Accordingly, the odds of having adequate counseling in the age group of 37–49 was almost three (AOR = 2.7; 95% CI: 1.1–6.6) times the odds of having adequate counseling in the age group of 19–24. Similarly, clients in the age group of 25–30 were 0.4 (AOR = 0.6; 95% CI: 0.4–0.9) times less likely to have adequately counseled compared with their counterparts.

Married clients were about three (AOR = 2.8; 95% CI: 1.2–6.7) times more likely to have adequate counseling compared with those who were single. Again, clients who attended secondary education level and higher educational level were almost 2 (AOR = 1.9; 95% CI: 1.1–3.6) and 1.9 (AOR = 2.2; 95% CI: 1.2–4.3) times more likely to have been adequately counseled than clients with no formal education, respectively**.** Finally, clients who had previous experience of attending family planning clinics were 1.6 (AOR = 1.6; 95% CI: 1.1–2.4) times more likely to have been adequately counseled than new clients (Table 4).

**Table 4:** Factors associated with inadequate family planning counseling among women attending family planning services at health facilities in Akaki Kality sub-city, Addis Ababa, Ethiopia, 2020.

Variables	Family planning counseling		COR (95% CI)	AOR (95% CI)	*p*-value
	Adequate counseling *N* (%)	Inadequate counseling *N* (%)			
Age					
19–24	81 (27.9)	209 (72.1)	1	1	
25–30	73 (24.9)	220 (75.1)	0.9 (0.6–1.2)	0.6 (0.4–0.9)	0.010^a^
31–36	26 (40.0)	39 (60.0)	1.7 (1.0–3.0)	1.7 (0.9–3.2)	0.123
37–49	17 (56.7)	13 (43.3)	3.4 (1.6–7.3)	2.7 (1.1–6.7)	0.029^a^
Educational level					
No formal education	29 (25.4)	85 (74.6)	1	1	
Primary education	60 (20.7)	230 (79.3)	0.8 (0.5–1.3)	0.8 (0.4–1.5)	0.459
Secondary education	68 (41.0)	98 (59.0)	2.0 (1.2–3.4)	1.9 (1.1–3.6)	0.039^a^
Higher education	40 (37.0)	68 (63.0)	1.7 (1.0–3.0)	2.2 (1.2–4.3)	0.016^a^
Marital status					
Single	15 (16.3)	77 (83.7)	1	1	
Married	182 (31.1)	404 (68.9)	3.0 (1.6–5.4)	2.8 (1.2–6.7)	0.020^a^
Number of children					
0	25 (19.4)	104 (80.6)	1	1	
1–2	155 (32.4)	323 (67.6)	2.0 (1.2–3.2)	1.0 (0.5–2.1)	0.995
3–4	17 (23.9)	54 (76.1)	1.3 (0.7–2.6)	0.5 (0.2–1.2)	0.116
Visit status					
New	65 (22.2)	228 (77.8)	1	1	
Revisit	132 (34.3)	253 (65.7)	1.8 (1.3–2.6)	1.6 (1.1–2.4)	0.028^a^
FP method used					
Pills	15 (25.4)	45 (74.6)	1	1	
Injectable	57 (28.5)	143 (71.5)	1.12 (0.6–2.3)	1.4 (0.7–2.8)	0.353
Implant	118 (29.8)	278 (70.2)	1.3 (0.7–2.3)	1.5 (0.8–2.8)	0.258
IUCD	7 (30.4)	16 (69.6)	1.3 (0.4–3.7)	1.1 (0.3–3.5)	0.874

CI, confidence interval; COR, crude odds ratio; AOR, adjusted odds ratio; FP, family planning IUCD, intrauterine devices.

^a^ Significantly associated at p-value < 0.05.

## Discussion

This study aims at assessing the quality of family planning counseling and its associated factors. Accordingly, the proportion of women who had inadequate family planning counseling was 70.9% (95% CI: 67.4%–74.3%). The age of respondents, marital status, educational status, and the number of children possessed were found to be significantly associated with the quality of family planning counseling.

The finding of this study revealed that only 29.1% (95% CI: 25.7%–32.6%) of the clients had adequate counseling. This finding is in line with a study conducted at St. Paul's Hospital and an Ethiopian national survey, which reported that the proportion of adequate family planning counseling during the postpartum period was 28.2% and 30%, respectively (18, 20). However, this finding is higher than a study conducted in health facilities in Senegal (18%) (20); this difference might be due to the difference in the tools used to assess the quality of family planning counseling.

On the other hand, the findings from this study are lower than studies conducted in Jordan and Iran; those studies reported that 42.9% and 38.9% of women attending family planning services were adequately counseled, respectively (19, 21). Also, the finding is much lower than a study conducted in Kenya (56.7%) (11). This difference might be due to differences in the quality of the health systems of the countries and socioeconomic differences.

Concerning specific elements of the standard framework of FP counseling, a significant difference was observed between this and other similar studies. In relation to making the client feel comfortable, none of the clients reported that a service provider introduced themselves to them. This finding is similar to that of the study conducted in Jordan and Port Said city (21, 22). In this study, 94.5% of the service providers maintained the privacy of the client. This is higher than the study conducted in St. Paul's Hospital during postnatal care (41.1%) (15). In this study, 69.3% of participants reported that the service providers greeted them. This is lower than the study conducted in maternal and child health centers in Port Said city 88.64% (22). This might be because of the differences in the client–provider ratio. In Ethiopia, the number of health professionals is very few compared with the population size (19), which results in the provider–client ratio being very low. In low-income countries, there is usually an insufficient human resource that cannot balance the healthcare need. This in turn makes a long waiting time. Thus, the professionals mainly focus on addressing a number of clients while overlooking the procedures. A study conducted in Malawi also reported that the lack of greetings was mainly due to insufficient human resources (23).

In the present study, only 22.4% of the counselors asked about the concerns of their clients regarding the use of modern FP methods. This is lower than the study conducted in St. Paul's Hospital (32.5%) (15). This might be explained by the difference in the levels of the facilities; St. Paul's Hospital is a specialized hospital while only health centers were included in this study. During the postpartum period, family planning counseling is very crucial and the most effective in reducing the unmet need for family planning (19). In this study, only 40% of clients were asked about childbearing intentions, which is lower than the study conducted in St. Paul's Hospital (99.5%) (15). The difference might be because of the differences in the study population; the study conducted in St. Paul's Hospital was conducted among postpartum women, while the current study was conducted on clients as they came for family planning services.

This study found that the educational status of the clients is associated with the quality of family planning counseling; clients with no formal education received inadequate counseling compared with secondary and higher educated clients, and this finding is similar to research conducted in different countries (15–17, 24). In general, educated women have more understanding regarding family planning, and they might need less information during counseling. However, from the perspective of easily understanding the information, the more the clients are educated, the more they understand the issue being discussed. Thus, more educated clients' counseling would be more successful than that of those who had no formal education at all.

Contrary to other studies conducted in Ethiopia (15–17), the findings from this study indicated that married clients received more adequate counseling than single clients. This might be explained by the service provider disapproving of the sexual activity of single women. This is evidenced by a study conducted in Ethiopia, where the providers might not provide family planning services with a restriction based on marital status (25).

In this study, older age is associated with adequate counseling. Evidence also indicated that adolescents in the low- and middle-income countries were found to receive poorer family planning counseling (19). However, a study in Texas City shows that older age was negatively associated with receiving postpartum counseling about Intrauterine Devices (IUDs) and implants (24). This difference might be because of the disparity in the study population; the study conducted in Texas included prenatal and postnatal women. Again, the majority of the study population was older in the study conducted in Texas contrary to the current study.

The result of this study showed returning clients had an adequate counseling than new clients, and this finding is contrary to studies conducted in Senegal and Iran (19, 20). This might be explained by the fact that returning clients have a better ability to understand what took place during counseling than new clients. It might also indicate that revisiting clients have a good client–provider relationship.

The findings of this study have implications for research since there is a paucity of studies in this area, and it helps the health centers to improve the service. Despite this, assessing the level of FP counseling only from the client's perspective and the data collected from interviews may be susceptible to recall bias. Even though the interview was conducted immediately after the counseling session, the client might not remember every detail of what took place. In addition, facility and health provider-related factors that might influence the quality of family planning counseling were not included in this study. Finally, the study was conducted in a specific sub-city of Addis Ababa; thus, the study might not be representative of the city.

## Conclusion

In conclusion, the counseling given to the clients was inadequate. The study also identified various factors that influence the quality of family planning counseling. These factors include clients' age, educational status, marital status, and visit status of clients. This implies that health professionals should give due attention to younger women, single clients, and clients with their first presentation to the health facility. It also indicates that promoting education among Ethiopian women is crucial for a positive outcome of family planning counseling.

## Data Availability

The raw data supporting the conclusions of this article will be made available by the authors, without undue reservation.
